# Laparoscopic surgery in elderly patients with sliding hiatal hernia

**DOI:** 10.1186/1471-2318-11-S1-A56

**Published:** 2011-08-24

**Authors:** R Scilletta, A Pesce, MA Trovato, A Branca, TR Portale, B Scilletta, S Puleo

**Affiliations:** 1 Department of General Surgery, University of Catania, AOU Policlinico-Vittorio Emanuele, Catania, Italy

## Background

Surgery is recommended as treatment for the sliding hiatal hernia (SHH) in order to create a barrier to reflux of gastric contents into the esophagus and into the upper airways where could cause aspiration pneumonia.

The aim of this study was to evaluate the results of laparoscopic surgery in elderly patients over 65 years, with SHH with typical and atypical respiratory symptoms, who have been followed up for 5 years.

## Materials and methods

235 patients with gastroesophageal reflux disease and SHH have been operated on from 2003 to 2010 at the Department of Surgical Sciences and Organ Transplantation of University of Catania. We conducted a study on 30 patients over 65 years belonging to that group.

Patients’ characteristics:

- Mean age 69 years, 22 females and 8 males

3. Typical and atypical symptoms in 18, in 12 typical

4. Reflux esophagitis grade II in 14, III in 6 , NERD in 10

- Radiography of the upper digestive tract: SHH in 29 patients with an average diameter of 5 cm, in 1 case 2 / 3 of the stomach in the chest.

3. Preoperative value:

4. LES pressure: 5.4 mmHg (range 3.8 to 8.1), LES length: 1 cm

- pHmetry median: 66.1

Surgery: Nissen Rossetti (NR) 360 ° fundoplication in 24; in 5 Toupet fundoplication 270° in patient with esophageal motility disorders, NR + Anterior gastropexy sec. Boherema in 1 case with a migration of the stomach into the chest. Section of short gastric vessels (SGV) in 16, and no section in 14.50 cc medium drainage, mean hospital stay: four days.

## Results

Postoperative transient dysphagia in 2 patients without section of SGV and in 1 with section. No permanent dysphagia. Resolution of symptoms in typical and atypical in 87% of 19 patients who agreed to perform postoperatively functional tests.

Mean postoperative LES pressure: 5 mmHg; LES length :3-4 cm; De Meester score: 8.4.

Resolution of esophagitis in 4, with no typical and atypical symptoms. Average 5 kg weight loss.

SHH recurrence in 2 patients at 3 years, recurrence of typical and atypical symptoms in 4, in 3 of these there were no section of the SGV.

## Conclusions

Surgery is the only therapeutic option able to create a mechanical barrier to reflux, that improves symptoms and stops both the development of Barrett’s esophagus on the underlying esophagitis and stops the digestive tract inhaling material. Surgery is, indeed, safe and effective and age does not represent a contraindication.

